# Coma With Absent Brainstem Reflexes and a Burst Suppression on EEG Secondary to Baclofen Toxicity

**DOI:** 10.3389/fneur.2020.00404

**Published:** 2020-05-13

**Authors:** Sahar Farhat, Tarek El Halabi, Achraf Makki, Samir F. Atweh, Wassim Nasreddine, Ahmad Beydoun

**Affiliations:** Department of Neurology, American University of Beirut Medical Center, Beirut, Lebanon

**Keywords:** coma, baclofen toxicity, burst suppression, brainstem reflexes, seizure

## Abstract

Baclofen, a muscle relaxant prescribed for the alleviation of symptoms of spasticity acts primarily at the spinal level but with high doses, it penetrates the blood-brain barrier and can result in prominent central nervous depression. Baclofen toxicity has been associated with a variety of symptoms ranging from dizziness to deep coma. We report the clinical course, management, and outcome of a case of baclofen overdose who presented in deep coma with loss of brainstem reflexes and a burst suppression (BS) pattern on his electroencephalogram (EEG). In addition, we reviewed the presentation and outcomes of all reported cases of baclofen toxicity with a BS pattern on EEG to evaluate if those cases share a common clinical presentation and for the presence of signs and symptoms that would help the clinician to consider this diagnosis. There appears to be a common clinical picture associated with severe baclofen toxicity consisting of deep coma associated with loss of all brainstem reflexes including pupillary reactivity, frequent association with seizures/myoclonic jerks, and a BS pattern on EEG. The outcome is generally good, and serial EEGs are recommended to document a reversal of the abnormal electrographic features.

## Introduction

Baclofen, [β-(4-chlorophenyl) GABA] is a skeletal muscle relaxant frequently prescribed for the alleviation of symptoms of spasticity (1), which is associated with various neurologic conditions such as multiple sclerosis and spinal cord lesions. It is also used for the treatment of other conditions such as chronic back pain associated with muscle spasms, trigeminal neuralgia, cluster headaches, or intractable hiccups (2). As a structural analog to gamma-aminobutyric acid (GABA), it is believed to exert its mechanism of action by binding to the GABA-B receptors at the level of the spinal interneurons (1). In therapeutic doses, baclofen primarily acts at the spinal level (3) and is rarely associated with severe adverse effects although drowsiness may occur (4). However, at higher doses it penetrates the blood-brain barrier and can result in prominent central nervous depression (5, 6).

Baclofen toxicity has been associated with a variety of symptoms ranging from dizziness to deep coma (7, 8). We report the clinical course, management, and outcome of a case of baclofen overdose who presented in deep coma with loss of brainstem reflexes and a burst suppression (BS) pattern on his electroencephalogram (EEG). In addition, we reviewed the presentation and outcomes of all reported cases of baclofen toxicity with a BS pattern on EEG to evaluate if those cases share a common clinical presentation and for the presence of signs and symptoms that would help the clinician consider this diagnosis.

## Case Report

A 68-year old right-handed man was brought to the emergency department (ED) with an altered level of consciousness. His present medical illness started a few hours prior to presentation with somnolence followed by unresponsiveness associated with multifocal myoclonus.

Upon admission, the patient required immediate intubation and ventilator support to protect his airway. On his initial examination, his blood pressure was 115/54 mm Hg, heart rate was regular at 54 beats/min and his temperature was 36.6°C. On neurological examination, the patient was in deep coma, with 4 mm fixed unreactive pupils, and absent corneal, oculocephalic, and gag reflexes. Funduscopic examination was normal, and neck was supple with no evidence of meningismus. There were no spontaneous movements with evidence of diffuse hypotonia and no response to noxious stimuli. The reflexes were diffusely reduced and the plantar responses were flexor bilaterally. The rest of his medical examination was normal with no external signs of a traumatic head injury. In the ED, the patient developed a generalized tonic-clonic seizure that was abolished with intravenous diazepam and was followed by a loading dose of valproate.

**Figure 1:**
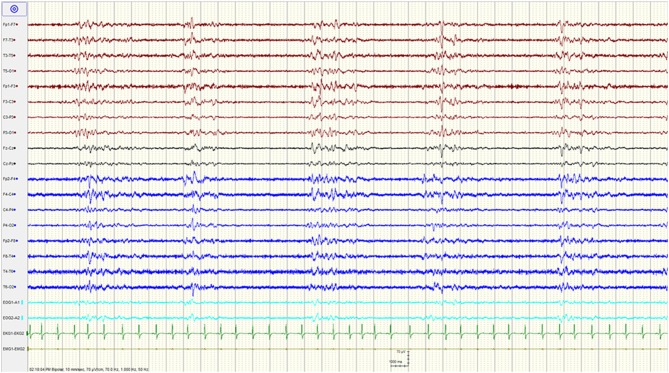
Burst suppression pattern upon presentation to the Emergency department.

A complete blood count and differential, electrolytes, liver function tests, renal function, and ammonia were unremarkable. Serum creatine kinase and venous lactic acid were elevated at 702 IU/L (reference range: 20–205 for males), and 2.27 mmol/L (reference range: 0.55–2.20), respectively. A urine toxicology screen was positive for benzodiazepines and opiates. A head computed tomography (CT) and a CT-angiogram were normal. Chest x-ray and electrocardiogram were normal. An epilepsy protocol magnetic resonance imaging (MRI) of the brain failed to reveal any abnormalities, specifically no restricted diffusion. A 90 min video/EEG recording in the ED showed a burst suppression pattern with bursts of mixed theta delta activity lasting 1–3 s alternating with severe diffuse suppression of the background amplitude lasting 4–5 s (Figure 1). There was no spontaneous variability in the record nor any reactivity to noxious or auditory stimuli.

His past medical history is relevant for a history of chronic low back pain associated with muscle spasms for which he was prescribed pregabalin, tramadol, and baclofen. Based on that history along with the BS pattern seen on EEG, all his home medications were held and blood for tramadol and baclofen serum levels were immediately drawn in the ED.

**Figure 2:**
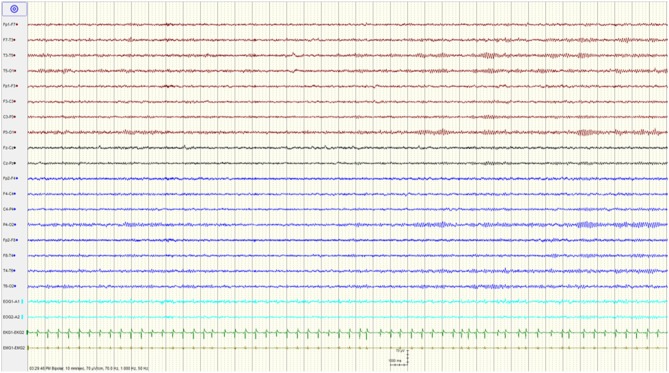
Resolution of the burst suppression pattern and normalization of the EEG after stopping baclofen.

The patient was admitted to the intensive care unit where his mental status gradually improved over the subsequent 48 h. He was extubated on day 3 at which time he was following commands, although he remained intermittently agitated, and required treatment with quetiapine. His EEG on that day showed resolution of the BS pattern with mild generalized slowing of the background (Figure 2). The patient was discharged home with no neurological deficits on day 9. The serum levels drawn on the day of admission revealed an elevated tramadol level at 1,960 ng/mL (Reference range: 100–1,000 ng/mL), and a toxic baclofen level at 4.30 ug/ml (Reference range 0.08–0.6 ug/ml; toxic >1.1ug/ml).

## Literature Review

We searched for all English-language reported cases of baclofen overdose via MEDLINE using the following key words: “baclofen,” “overdose,” “intoxication,” “EEG,” and “burst suppression.” Additional studies were obtained by checking the references. We reviewed all reported cases of accidental or intentional oral baclofen overdose to determine if a BS pattern was present on the EEG. The nine cases identified from the literature (including the one reported here) are listed in Table 1, along with some of the findings on neurological examination and pertinent clinical features.

**Table 1:** Clinical characteristics of published cases of baclofen intoxication with a BS pattern on EEG.

**References**	**Age (yrs)**	**Gender**	**Presentation**	**Intubated**	**Brainstem reflexes**	**Pupillary reflexes**	**Reflexes**	**Seizure**	**Myo-clonus**	**EEG pattern**	**Baclofen level (ug/ml)**	**Start of recovery**
Paulson (9)	29	F	Deep coma	Yes	Absent	Absent	NR	No	Yes	BS	ND	Day 3
Weissenborn et al. (10)	40	F	Deep coma	Yes	Absent	Absent	Absent	Yes	No	BS	1.2	Day 4
Ostermann et al. (11)	59	M	Deep coma	Yes	Absent	Absent	Hypo	No	No	BS	ND	Day 2
Slaughter et al. (12)	14	F	Deep coma	Yes	Absent	Absent	Absent	Yes	No	BS	0.6	Day 4
Wall et al. (13)	48	M	Deep coma	Yes	NR	Absent	NR	Yes	No	BS	1.2	Day 2
Kumar et al. (14)	35	F	Deep coma	Yes	Absent	Absent	Absent	No	Yes	BS	ND	Day 3
Sullivan et al. (15)	40	F	Deep coma	Yes	Absent	Absent	NR	Yes	No	BS	ND	Day 5
Caron et al. (2)	17	F	Deep coma	Yes	Absent	Absent	Absent	Yes	No	BS	0.8	Day 5
Our case	68	M	Deep coma	Yes	Absent	Absent	Hypo	Yes	Yes	BS	4.3	Day 3

NR, Not reported; ND, Not done; Hypo, hyporeflexia; M, Males; F, Females.

*Start of recovery indicated the time when patient was extubated and started to follow commands*.

## Discussion

This case demonstrates that severe baclofen toxicity with a BS pattern on EEG is fully reversible with adequate supportive treatment. The baclofen overdose was documented with a serum level drawn shortly after presentation to the ED and was ~7 times the upper limit of the reference range and four times higher than the toxic level.

A series of 37 patients reported that the common clinical presentations of baclofen overdose consisted of an encephalopathy in all patients, coma in 59%, seizures in 43%, as well as respiratory depression, hyporeflexia, and autonomic dysfunction (7). Of the 16 patients with seizures, 50% experienced generalized tonic-clonic seizures, 31% experienced myoclonus, and 19% experienced both seizure types. Other less frequent manifestations included hallucinosis, impaired memory, catatonia, acute mania, orofacial dyskinesia, and tremor (7). The severity of symptoms with baclofen toxicity were found to be dose-related with delirium, seizures, and coma only seen following ingestion of 200 mg or more of baclofen (8).

Baclofen toxicity is also associated with a variety of EEG abnormalities including generalized slowing of the background activity, appearance of triphasic waves, generalized periodic sharp waves, or rhythmic generalized high-amplitude delta waves (16–19). A BS pattern, which consists of bursts of mixed activity alternating with severe suppression of the background amplitude has rarely been reported following baclofen intoxication. This pattern, which indicates a severe cerebral dysfunction and depressed function of the deep pontine areas (20) is mostly seen after diffuse hypoxic-ischemic injuries in which case it portends a very bad prognosis (21). It was also reported after severe traumatic brain injuries and following prolonged status epilepticus (10). When associated with a toxic etiology, this pattern can be reversible with the patient making a complete recovery. Intoxications leading to a BS pattern are rare and have mostly been described after barbiturates or gluthetimide overdoses (22). In our patient, although the level of tramadol was above the therapeutic range and could have contributed to his encephalopathy, intoxication with this drug was never reported to lead to a BS pattern. The electrographic features associated with tramadol intoxication mostly consisted of epileptiform and non-epileptiform abnormalities with no BS pattern reported (23–25). Similarly, the clinical features of pregabalin toxicity described in a few case reports consisted of patients presenting in a comatose state, severe neurological depression, encephalopathy, or psychosis associated with rhythmic epileptiform discharges or triphasic waves over the scalp EEG (9, 26, 27).

The first case of baclofen intoxication associated with a BS pattern on EEG was published in 1976 and described a 29 years old woman who ingested 900–970 mg in a suicide attempt (11). Since then, seven additional cases of accidental or intentional intoxication with baclofen associated with a BS pattern on EEG have been reported (Table 1). Upon review of those cases, which included adults and children, it is clear that the initial clinical presentation is very similar and consists of deep coma, absence of brainstem reflexes including pupillary reflexes, respiratory failure, and diffuse hypotonia with hyporeflexia or areflexia (Table 1). This initial clinical presentation can therefore suggest a catastrophic central nervous system (CNS) event, sometimes mimicking brain death (15, 28). A striking clinical feature of all reported cases (including our own) is the absence of pupillary reflexes which are usually preserved in metabolic encephalopathies and most drug toxicities, a finding that would favor a severe hypoxic, or traumatic injury, or a brainstem lesion (29). However, there was no consistent pattern regarding the pupillary size since out of 9 patients, 5 patients were reported to have midsize pupils, two had dilated pupils, one had small pupils, and the pupillary size in the last patient was not reported. A severe anoxic or traumatic injury as well as intoxication with certain drugs can lead to a BS pattern on EEG, a pattern that would not be seen following an ischemic event or a lesion to the brainstem.

In addition, there was a frequent association with generalized tonic-clonic seizures or multifocal myoclonic jerks as five of the nine patients presented with generalized tonic-clonic seizures, two with myoclonus, and one patient experienced both seizure types (Table 1). Although an analog of GABA, an inhibitory neurotransmitter, baclofen interacts with the GABA-B receptors that are located on pre and postsynaptic positions. It was therefore suggested that baclofen can act as a proconvulsant by hyperpolarizing presynaptic and postsynaptic inhibitory interneurons, which will shift the neuronal balance toward excitation and lower the seizure threshold (30).

The time span for recovery is overall consistent with the half-life reported for baclofen overdose. Although with chronic dosing the elimination half-life of baclofen was estimated at 3–4 h (31), it was reported to sometimes increase to more than 30 h following ingestion of toxic doses (32). In addition, experimental studies have shown that its elimination from the CNS is slower than its pharmacokinetic half-life (33). This can explain the persistence of CNS depression even when the plasma concentrations of baclofen levels return to within the therapeutic range (14, 32).

Since there is no antidote for baclofen, the management of overdose is symptomatic and consists of supportive care with intravenous fluids, inotropes, and mechanical ventilation when necessary (15, 34). All reported patients who presented in deep coma and a BS pattern on EEG required intubation, were admitted to the intensive care unit (ICU) for a few days and recovered completely with adequate supportive care (Table 1). Although not an antagonist to baclofen, some have advocated the use of physostigmine in cases of baclofen toxicity based on the fact that this drug reversed the respiratory depression and somnolence due to opiates (35). However, its lack of efficacy as reported by others coupled with its adverse effects that include bradycardia and increased airway secretions would argue against its routine use (36).

Others have suggested the use of flumazenil, a benzodiazepine antagonist (37), but subsequent reports failed to support its use in cases of baclofen toxicity (38). Hemodialysis can be considered especially in patients with renal insufficiency, since it will result in a delayed clearance of baclofen (7, 39).

## Conclusions

Since baclofen is a commonly prescribed drug and is also used as recreational drug [38], it is important for clinicians to be aware of the signs and symptoms associated with an overdose, whether accidental or intentional. There appears to be a common clinical picture associated with severe baclofen toxicity consisting of deep coma associated with loss of all brainstem reflexes including pupillary reactivity, frequent association with seizures/myoclonic jerks, and a BS pattern on EEG. When hypoxic or traumatic injuries are ruled out, this clinical picture should strongly suggest the possibility of intoxication and a serum level of baclofen, when possible, should be drawn. A high level of suspicion is required since baclofen is not detected by routine urine drug screening. The outcome is generally good, and serial EEGs are recommended to document a reversal of the abnormal electrographic features.

## Data Availability Statement

The raw data supporting the conclusions of this article will be made available by the authors, without undue reservation, to any qualified researcher.

## Ethics Statement

Written informed consent was obtained from the participant for the publication of this case report.

## Author Contributions

AB, AM, and SA contributed to the conception of the case. SF and TE collected the data and wrote the manuscript. AB and WN edited the manuscript. All the authors contributed to manuscript revision, read, and approved the submitted version.

## Conflict of Interest

The authors declare that the research was conducted in the absence of any commercial or financial relationships that could be construed as a potential conflict of interest.
